# Application of Convolutional Neural Networks in the Diagnosis of *Helicobacter pylori* Infection Based on Endoscopic Images

**DOI:** 10.1016/j.ebiom.2017.10.014

**Published:** 2017-10-16

**Authors:** Satoki Shichijo, Shuhei Nomura, Kazuharu Aoyama, Yoshitaka Nishikawa, Motoi Miura, Takahide Shinagawa, Hirotoshi Takiyama, Tetsuya Tanimoto, Soichiro Ishihara, Keigo Matsuo, Tomohiro Tada

**Affiliations:** a Tada Tomohiro Institute of Gastroenterology and Proctology, Japan; b Department of Gastrointestinal Oncology, Osaka International Cancer Institute, Japan; c Department of Global Health Policy, Graduate School of Medicine, The University of Tokyo, Japan; d Department of Epidemiology and Biostatistics, School of Public Health, Imperial College London, UK; e Idee, Inc., Japan; f Department of Health Informatics, Kyoto University School of Public Health, Japan; g Teikyo University of Graduate School of Public Health, Japan; h Department of Surgical Oncology, Graduate School of Medicine, The University of Tokyo, Japan; i Medical Governance Research Institute, Japan; j Jyoban Hospital of Tokiwa Foundation, Japan; k Surgery Department, Sanno Hospital, International University of Health and Welfare, Japan; l Department of Gastroenterology, Tokatsu-Tsujinaka Hospital, Japan

**Keywords:** *Helicobacter pylori*, Endoscopy, Artificial intelligence, Convolutional neural networks

## Abstract

**Background and aims:**

The role of artificial intelligence in the diagnosis of *Helicobacter pylori* gastritis based on endoscopic images has not been evaluated. We constructed a convolutional neural network (CNN), and evaluated its ability to diagnose *H. pylori* infection.

**Methods:**

A 22-layer, deep CNN was pre-trained and fine-tuned on a dataset of 32,208 images either positive or negative for *H. pylori* (first CNN). Another CNN was trained using images classified according to 8 anatomical locations (secondary CNN). A separate test data set (11,481 images from 397 patients) was evaluated by the CNN, and 23 endoscopists, independently.

**Results:**

The sensitivity, specificity, accuracy, and diagnostic time were 81.9%, 83.4%, 83.1%, and 198 s, respectively, for the first CNN, and 88.9%, 87.4%, 87.7%, and 194 s, respectively, for the secondary CNN. These values for the 23 endoscopists were 79.0%, 83.2%, 82.4%, and 230 ± 65 min (85.2%, 89.3%, 88.6%, and 253 ± 92 min by 6 board-certified endoscopists), respectively. The secondary CNN had a significantly higher accuracy than endoscopists (by 5.3%; 95% CI, 0.3–10.2).

**Conclusion:**

*H. pylori* gastritis could be diagnosed based on endoscopic images using CNN with higher accuracy and in a considerably shorter time compared to manual diagnosis by endoscopists.

## Highlights

• We compared the diagnostic ability for *H. pylori* gastritis between a convolutional neural network (CNN) and endoscopists.
• Diagnostic ability of CNN was greater than that of endoscopists in general and similar to that of experienced endoscopists.
• The diagnostic time of CNN was considerably shorter than that of manual diagnosis by endoscopists.

In Japan, *H. pylori* infection is common, and its detection during endoscopic examination is desirable. However, a diagnosis based on endoscopic findings requires training, and its accuracy depends on the endoscopist's skill. We showed that the diagnostic ability of a convolutional neural network (CNN) for *H. pylori* infection was comparable to that of experienced endoscopists, and the time required for the diagnosis was considerably shorter. Thus, the use of CNNs for diagnosis of *H. pylori* infection will reduce endoscopists' workload. Moreover, the procedure can be performed completely “online,” thereby addressing the problem of inadequate number of physicians in remote locations.

## 1 Introduction

Gastric cancer is one of the most common malignancies, with one million cases estimated around the world in 2012 (O'Connor et al., 2017). Among the underlying causes, *Helicobacter pylori* (*H. pylori*) infection plays a central role in the pathobiology of gastric cancer; it induces atrophic gastritis and intestinal metaplasia, eventually resulting in the development of gastric cancer (Correa, 1995, Correa and Houghton, 2007, Take et al., 2015, Shichijo et al., 2015). Given the increased risk of gastric cancer in *H. pylori*-infected patients, and the decreased incidence of gastric cancer following *H. pylori* eradication, the International Agency for Research on Cancer has categorized *H. pylori* as a definite carcinogen (Ogura et al., 2008, Fukase et al., 2008, Ford et al., 2014, Yoon et al., 2014).

An endoscopic examination is often performed for the screening of gastric cancer and other diseases. It is also useful for the detailed examination of various epigastric symptoms, positive barium meal studies for gastric diseases, and abnormal serum pepsinogen levels. Additionally, an endoscopic examination is helpful in diagnosing *H. pylori* infection; atrophy, diffuse redness, mucosal swelling, enlarged folds, and nodularity are representative findings for *H. pylori*-positive gastritis, while a regular arrangement of collecting venules and fundic gland polyps are characteristic of *H. pylori*-negative gastric mucosa (Kato, 2016). A precise endoscopic diagnosis of *H. pylori* infection will trigger confirmation by various tests such as blood or urine anti-*H. pylori* IgG levels, fecal antigen test, urease breath test, or rapid urease test. Subsequently, patients with a positive test result are considered for *H. pylori* eradication therapy for the prevention of gastric cancer and other diseases, which are covered by national health insurance in Japan.

However, a diagnosis based on endoscopic findings requires training (Sugano et al., 2015, Watanabe et al., 2013), is time-consuming and subjective, and may result in false-positive and false-negative results depending on the skill of the endoscopist. Further, fatigue may adversely affect the diagnostic yield of this investigation as shown in a previous report, wherein the adenoma detection rates via colonoscopy declined with increasing procedural hours (Almadi et al., 2015).

Recent reports suggest a role for artificial intelligence (AI) using deep learning in various medical fields, especially as a system with the ability to screen medical images, in areas including radiation oncology (Bibault et al., 2016), skin cancer classification (Esteva et al., 2017), and diabetic retinopathy (Gulshan et al., 2016). In the context of medical imaging, deep learning has the potential to become a powerful machine learning technique that can interpret medical images based on a set of unique algorithms developed by historically accumulated data (LeCun et al., 2015). Deep learning allows computational models that are composed of multiple processing layers to learn representations of data with multiple levels of abstraction (LeCun et al., 2015).

The convolutional neural network (CNN) has been developed by Szegedy et al., and is the most popular network architecture for deep learning for images. To evaluate whether CNN has a role in identifying *H. pylori* infection based on endoscopic images, we constructed an AI-based diagnostic system that was trained using > 30,000 endoscopic images. We tested this system by comparing its diagnostic accuracy for *H. pylori* gastritis with that of endoscopists.

## 2 Methods

### 2.1 Esophagogastroduodenoscopy Procedures

**Fig. 1:**
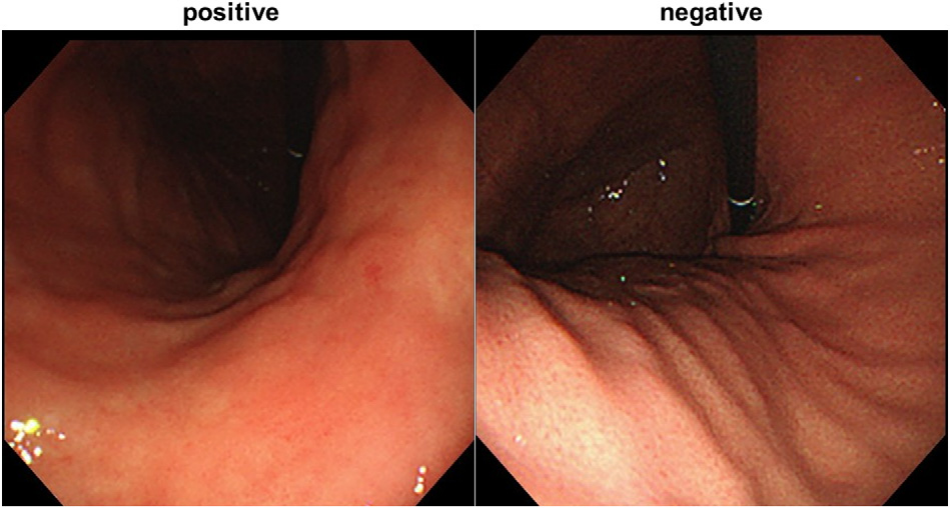
Representative endoscopic images of *Helicobacter pylori*-positive, and –negative stomach. Atrophy and diffuse redness are seen in the presence of infection. A regular arrangement of collecting venules (RAC) is seen in the uninfected stomach.

Thirty-three endoscopists performed esophagogastroduodenoscopy (EGD) at Tada Tomohiro Institute of Gastroenterology and Proctology (Saitama, Japan). The indications for EGD were referral from a primary care physician for evaluation of epigastric symptoms, positive results from gastric disease screening by barium meal, abnormal serum pepsinogen levels, a previous history of gastroduodenal disease, or as a part of routine screening for gastric cancer. Patients who received *H. pylori* eradication therapy were excluded from the current study. We performed standard EGD (EVIS GIF-XP290N, GIF-XP260, GIF-XP260NS, GIF-N260; Olympus Medical Systems, Co., Ltd., Tokyo, Japan) and captured esophagogastroduodenal mucosal images. Fig. 1 shows the typical images obtained by us. We did not use magnified images in this study.

### 2.2 Clinical Diagnosis of *H. pylori* as Reference Standard

All patients were tested for *H. pylori* infection by at least one of the following tests; blood or urine anti-*H. pylori* IgG levels, fecal antigen test, or urease breath test. Patients who tested positive on any of these assays were classified as *H. pylori*-positive.

### 2.3 Development Data Preparation

We prepared a data set (development data set) that was used to educate and construct the AI-based diagnostic system. The images of EGD performed for 1750 patients from January 2014 to December 2016 were retrospectively reviewed. Patients with the presence or the history of gastric cancer, ulcer, or submucosal tumor were excluded from the development data set. The endoscopic images of the stomach diagnosed as *H. pylori*-positive or *H. pylori-*negative, were further screened by endoscopists to exclude images that were unclear owing to various reasons, including food residue in the stomach, bleeding following biopsy, and halation. Finally, 32,208 images from patients that were classified as *H. pylori*-positive (735 patients) or negative (1015 patients) were prepared for the development data set (Table 1).

**Table 1:** Baseline characteristics.

Characteristics	Development data set	Test data set
No. of images	32,208	11,481
No. of endoscopists	33	13
No. of patients	1768	397
Age, mean (SD), y	52.7 (13.2)^a^	50.4 (11.2)
Sex, No. (%)		
Male	480 (45)^a^	168 (43)
Female	598 (55)^a^	226 (57)
*H. pylori* status, No. (%)		
Positive	753 (43)	72 (18)
Negative	1015 (57)	325 (82)

SD, standard deviation.

a Data were available for 1078 cases.

The 32,208 original endoscopic images for development were randomly rotated between 0 and 359°, their black frames were cropped, and the images were zoomed in/out on a scale of 0.9–1.1. Subsequently, they were augmented by a factor of 15. Blurred images were also used in the development dataset during training.

First, we constructed the CNN using all the images together. Second, we constructed the other CNN using the images classified according to 8 different locations in the stomach (cardia, upper body, middle body, lesser curvature, angle, lower body, antrum, and pylorus).

### 2.4 Test Data Preparation

**Fig. 2:**
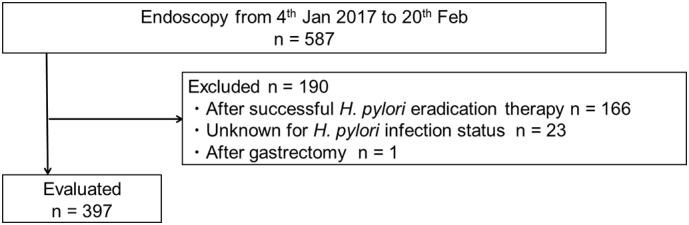
Patient recruitment flowchart.

To evaluate the diagnostic accuracy of the constructed CNN, and to compare it with endoscopists, a separate test data set was prepared. Among 587 patients who underwent endoscopic examination at the Tada Tomohiro Institute of Gastroenterology and Proctology from January to February 2017, 190 patients were excluded for various reasons: completed *H. pylori* eradication, 166; unknown *H. pylori* infection status, 23; and underwent gastrectomy, 1. Finally, the test data set included a total of 11,481 images from 397 patients (72 *H. pylori* positive, and 325 negative, respectively) (Fig. 2). Patient demographics and image characteristics are shown in Table 1. The diagnosis was established by a fecal antigen test in 172 (43%), and urine anti-*H. pylori* IgG levels in 87 (21%). There was no overlap between the test and the development datasets.

### 2.5 Training Algorithm

To construct an AI-based diagnostic system, we used a state-of-the-art deep neural network architecture, GoogLeNet (https://arxiv.org/abs/1409.4842), which had been developed by Szegedy et al. GoogLeNet is a deep CNN that consists of 22 layers. A Caffe deep learning framework, one of the popular and most widely used frameworks that was originally developed at the Berkeley Vision and Learning Center (BVLC), was then used to train, validate, and test the CNN.

**Fig. 3:**
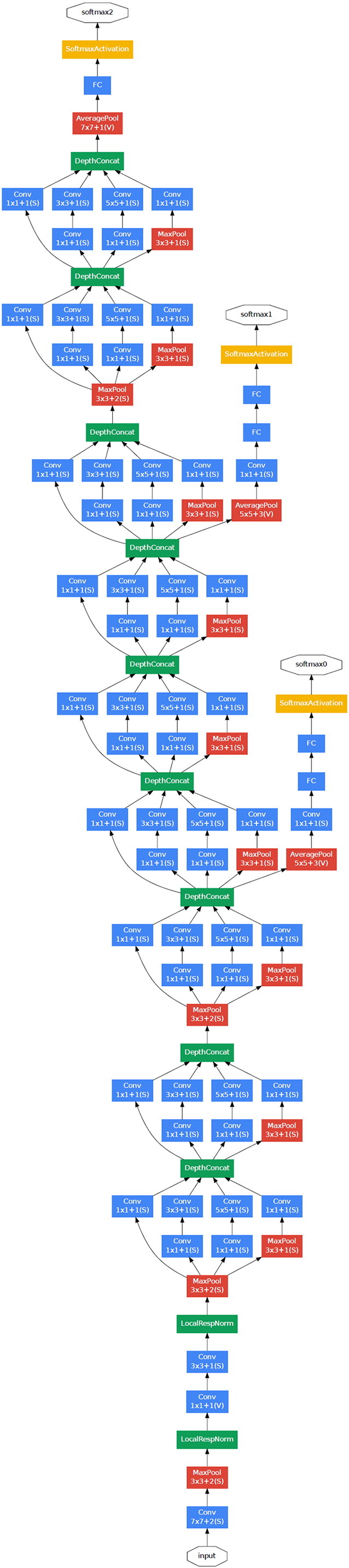
Deep convolutional neural network (CNN) layout. We used a CNN technique for image classification. Data flow is from bottom to top direction. With a given input image, the CNN architecture produces a probability distribution over classes as *H. pylori* positive or negative. The GoogLeNet, a deep CNN of 22 layers, is pre-trained on the ImageNet dataset and fine-tuned on our own dataset of about 400,000 endoscopic images, which are pre-augmented. GoogLeNet architecture published from https://arxiv.org/abs/1409.4842.

The deep CNN was trained using backpropagation (Fig. 3), a method of training neural networks, by which loss gradients for all the weights in the network can be computed efficiently. All layers of the network were fine-tuned by using Adam (https://arxiv.org/abs/1412.6980), a method for stochastic optimization with a global learning rate of 0.0001. To optimize our images for GoogLeNet, they were resized to 244 × 244 pixels. We used a pre-trained model that learned natural-image features through ImageNet. This procedure, known as transfer learning, is useful even with sparse training data.

### 2.6 Evaluation Algorithm

The trained neural network generated a continuous number between 0 and 1 for *H. pylori* positive or negative, corresponding to the probability of that condition being present in the image. Receiver operating curves (ROC) were plotted by varying the operating threshold.

### 2.7 Performance Comparison Between CNN and Endoscopists on Test Data Sets

The test endoscopic images were classified by the CNN, and 23 endoscopists of varying experience as *H. pylori*-positive or negative, in the absence of any other prior information. Six of the 23 endoscopists were Board Certified Gastroenterologists of the Japanese Gastroenterological Endoscopy Society (certified group). The other 17 endoscopists were further classified as: the “relatively experienced group”, having performed > 1000 EGDs (*n* = 9); and the “beginner group”, having performed < 1000 cases (*n* = 8). The sensitivity, specificity, and accuracy of *H. pylori* diagnosis of the CNN and the endoscopists were measured, and compared by using a two-tailed two sample proportions test.

The ROC for the diagnostic accuracy of CNN was described by using the R software. We used STATA/MP version 14.2 for all statistical analyses, and a *p*-value of < 0.05 was considered statistically significant.

All patient information was de-identified prior to the data analyses for maintaining patient anonymity. Patient details were not accessible to any of the endoscopists involved in the study. This study was approved by the Institutional Review Board of the Japan Medical Association (ID JMA-IIA00283), and conducted under the Declaration of Helsinki.

## 3 Results

### 3.1 Performance of Convolutional Neural Network

The CNN constructed in this study provided an output of the probability of *H. pylori* infection per image. This was followed by the algorithm calculating a mean square of the probabilities per patient. First, we examined the performance of the CNN constructed with unclassified images of the stomach. The area under the curve (AUC) for the ROC was 0.89. At a cut off value of 0.43, the value for which the point on the ROC curve corresponds to 100% sensitivity and specificity, the sensitivity, specificity, and accuracy of the CNN were 81.9% (95% confidence interval [CI], 71.1–90.0), 83.4% (95% CI, 78.9–87.3), and 83.1% (95% CI, 79.1–86.7), respectively. The diagnostic time for analyzing all the images by the CNN was 3 min and 18 s. For the 67 cases of “wrong diagnosis” attributed to the CNN, the average accuracy by the 23 endoscopists was 57.6% (standard deviation [SD], 33.2).

Next, we examined the performance of the other CNN constructed with images classified by their location in the stomach, and found that the AUC increased to 0.93 (Fig. 5). At a cutoff point of 0.34, the sensitivity, specificity, and accuracy of this CNN were 88.9% (95% CI, 79.3–95.1), 87.4% (95% CI, 83.3–90.8), and 87.7% (95% CI, 84.0–90.7), respectively. The diagnostic time for analyzing all the images by this CNN was 3 min and 14 s. The diagnosis was accurate for 348 cases out of 397, and the average endoscopist-accuracy for the 49 cases misdiagnosed by the CNN was 46.3% (SD, 34.9).

### 3.2 Performance of Endoscopists

Table 2 shows the results of image evaluation of the test data by the 23 endoscopists. The overall sensitivity, specificity, and accuracy for the diagnosis of *H. pylori* infection were 79.0% (SD, 11.7), 83.2% (SD, 9.8%), and 82.4% (SD, 8.4%), respectively. The average diagnostic time to evaluate all the images of the test data sets was 230.1 (SD, 65.0) min. The board-certified group was found to have significantly higher specificity (89.3% vs. 76.3%, *p* < 0.001) and accuracy (88.6% vs. 75.6%, p < 0.001) than the beginner group. Similarly, a significant difference in the specificity (85.1% vs. 76.3%, p < 0.001) and accuracy (84.4% vs. 75.6%, *p* < 0.05) was observed between the relatively experienced group and the beginner group.

**Table 2:** Diagnostic accuracy: CNN vs. endoscopists.

	CNN		Endoscopists			
	First CNN	Secondary CNN	Certified	Relatively experienced	Beginner	Total
No. of endoscopists			6	9	8	23
Sensitivity (SD), %	81.9	88.9	85.2 (4.5)	81.0 (10.2)	72.2 (14.3)	79.0 (11.7)
Specificity (SD), %	83.4	87.4	89.3 (2.6)	85.1 (8.7)	76.3 (10.8)	83.2 (9.8)
Accuracy (SD), %	83.1	87.7	88.9 (2.9)	84.4 (7.1)	75.6 (8.2)	82.4 (8.4)
AUC	0.89	0.93				
Time (SD), min	3.3	3.2	252.5 (92.3)	236.1 (51.9)	206.6 (54.7)	230.1 (65.0)

SD, standard deviation; AUC, area under the receiver operating curve.

### 3.3 Comparison Between CNN and Endoscopists

**Fig. 4:**
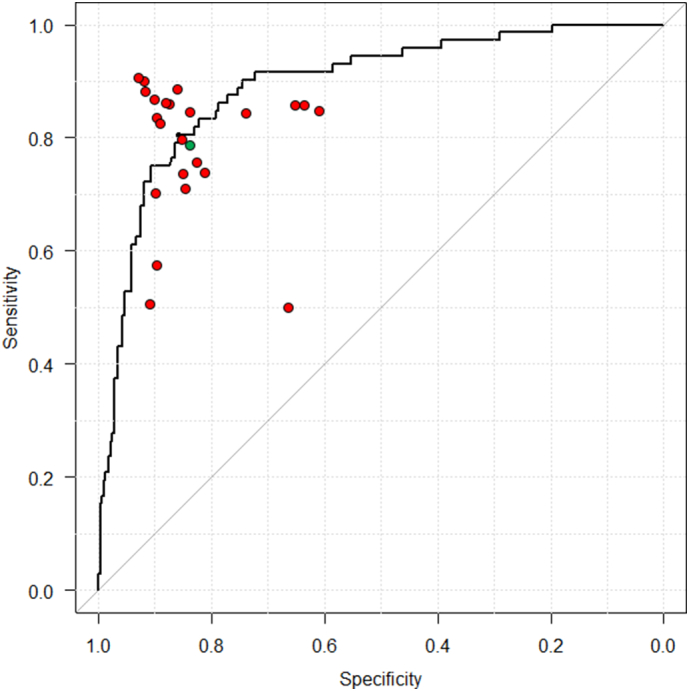
Receiver operating curves for CNN trained by uncategorized data and prediction of the endoscopists. Each endoscopist's prediction is represented by a single red point. The green point is the average prediction of the endoscopists. The CNN outputs a *H. pylori* probability P per image, and then the program calculates a mean square of the probabilities per patient. The area under the receiver operating curve is over 89%.

**Fig. 5:**
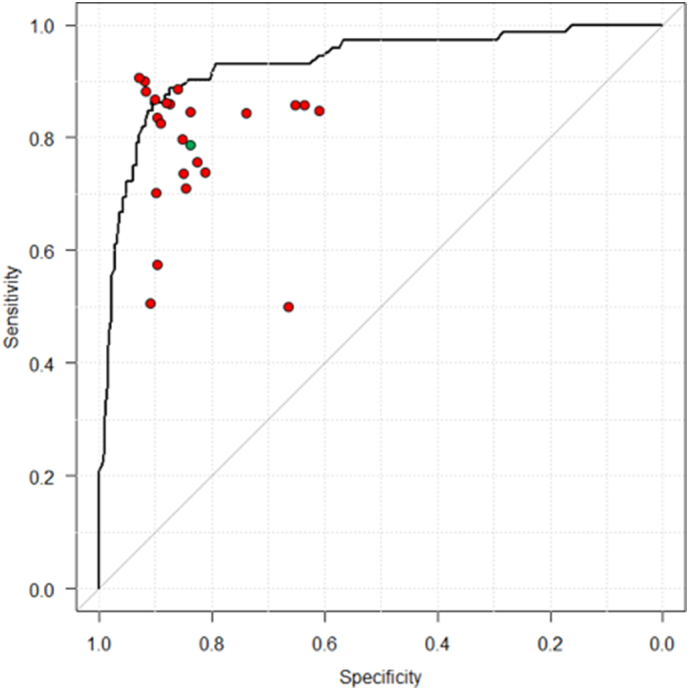
Receiver operating curves for CNN trained by categorized data. The CNN output demonstrates better probability following a training based on location-based classification of images. The area under the receiver operating curve is now 93%. Each endoscopist's prediction is represented by a single red point. The green point is the average prediction of the endoscopists.

At a cutoff point of the operating threshold of 0.43, and an AUC of 0.89, the CNN constructed with the unclassified images of the stomach was not statistically different from the 23 endoscopists in terms of its sensitivity, specificity, and accuracy (Fig. 4). At a cutoff point of 0.34, and an AUC of 0.93, the secondary CNN, constructed with images classified according to their location in the stomach, was found to have a significantly higher accuracy than the endoscopists (by 5.3%; 95% CI, 0.3–10.2, Fig. 5), although their sensitivity and specificity were comparable.

## 4 Discussion

We constructed a CNN algorithm for the diagnosis of *H. pylori* gastritis based on the analysis of endoscopic images and compared its diagnostic accuracy with that of endoscopists. The diagnostic ability of the CNN appeared to be comparable to that of experienced endoscopists. Additionally, the diagnostic time with the CNN was considerably shorter than with the endoscopists. Our results indicate that the screening system developed by us based on the CNN has adequate sensitivity and specificity for it to be introduced into clinical practice, and it has the potential to markedly reduce the workload of endoscopists.

In Japan, *H. pylori* infection is prevalent, especially among the elderly. The endoscopic mass screening for gastric cancer that was started in 2016 has resulted in a large volume of endoscopic images to be processed, necessitating a more efficient method of screening the images. The results of our study suggest that an automated analysis of these stored images by using the CNN developed by us can effectively screen for *H. pylori* infection and aid in identifying cases that need a confirmatory test.

It should be noted that the shorter screening time, and the absence of fatigue with CNN, may enable the provision of results immediately following the endoscopic examination. Further, the diagnosis of *H. pylori* infection by the CNN can be performed completely “online,” and may contribute to the incorporation of endoscopy reporting as a part of “telemedicine,” thereby addressing the problem of inadequate numbers of doctors in remote and distant locations.

Recently, deep learning algorithms for the detection of skin cancer and diabetic retinopathy have been reported (Esteva et al., 2017, Gulshan et al., 2016). The sensitivity and specificity of CNNs in diagnosing skin cancer were > 90%, while their ability to diagnose retinopathy was comparable to that of ophthalmologists. Unlike the skin or retina, the stomach is complex in its form, and endoscopy images are acquired from different parts of the stomach, including the cardia, body, angle, and pylorus. This may make the discrimination and interpretation of images by a CNN difficult. The conversion of three dimensional structures into two dimensional images may alter the interpretation of such images by endoscopists as well as CNN. Therefore, the construction of a deep learning algorithm for diagnosing *H. pylori* gastritis based on endoscopic images was considered to be difficult. To solve this problem, we successfully constructed a secondary CNN by using images that were classified according to their location in the stomach.

The gastric mucosal changes caused by *H. pylori* infection such as atrophy and intestinal metaplasia initially occur at the distal stomach (antrum), and gradually expand to involve the proximal stomach (corpus). As such, in those stomachs with mild changes limited to the antrum, a diagnosis based on the normal mucosa of the corpus may result in a misdiagnosis. Endoscopists reach a diagnosis after identifying the location of the stomach in the image and correlate the mucosal changes therein. We demonstrated that training the CNN by using images classified according to their anatomical locations in the stomach resulted in an increase in sensitivity from 81.9% to 88.9%, and improved its ability to a level matching that of the board-certified endoscopists.

There are several future possibilities in the AI-based diagnosis of *H. pylori* infection. Our study included archived images obtained by nasal endoscopes that have lesser information compared to images acquired by transoral endoscopes or with real-time imaging. In addition, there are reports detailing the diagnosis of *H. pylori* gastritis by image enhanced endoscopy (Dohi et al., 2016) or magnifying endoscopy (Kanzaki et al., 2012, Anagnostopoulos et al., 2007, Yagi et al., 2014). The use of such advanced technology may improve the diagnostic accuracy for humans as well as for CNN. It is interesting to estimate the improvement in the diagnostic ability of the CNN in combination with more advanced techniques. Further, the role of real-time diagnosis by CNN based on “live” images during the endoscopic examination also needs to be explored. We did not include patients that underwent *H. pylori* eradication in this study. We plan to construct a CNN for diagnosing patients following *H. pylori* eradication in a future study as a means of assessing the success of *H. pylori* eradication.

There are several limitations in this study. First, the development data set as well as the test data set were obtained from a single center. Validation by using images obtained at other facilities, and other endoscopy devices and techniques may enhance the generalizability of our results; however, we used more than ten thousand images in this study, and that may overcome this limitation. Second, the tests used to confirm the diagnosis of *H. pylori* infection status, and blood or urine anti-*H. pylori* IgG levels, as well as fecal antigen tests or urease breath tests, are not 100% sensitive or specific (Kato et al., 2000, Murakami et al., 2011, Sato et al., 2012, Ferwana et al., 2015). This may have influenced our assessment of the diagnostic ability of the CNN. This limitation may be overcome by providing information related to the method of confirming *H. pylori* infection status in the construction design of the CNN. Third, *H. pylori* infection status was confirmed in most patients using only one tests. However, by excluding patients after *H. pylori* eradication, who sometimes still harbor antibodies, and adding confirmation tests when experienced board certified endoscopists had doubt about the first tests results, the possibility of a false positive or negative *H. pylori* diagnosis was considered negligible.

In conclusion, the accuracy of the CNN was comparable to that of endoscopists in diagnosing *H. pylori* infection based on endoscopic images of the stomach. CNNs may aid in screening for *H. pylori* infection at a substantially shorter time and contribute to reducing the workload of endoscopists. Further research should be conducted for validation and widespread application of the CNN.
